# Non-linear association of the alanine aminotransferase to high-density lipoprotein cholesterol ratio with non-alcoholic fatty liver disease: a secondary analysis of a Chinese cohort

**DOI:** 10.3389/fmed.2025.1713878

**Published:** 2025-12-04

**Authors:** Xiaoqing Lin, Yaoxuan Peng, Junjie Huang, Ziyang Huang

**Affiliations:** 1 Department of Ultrasonography, Shantou Central Hospital, Shantou, Guangdong, China; 2 Department of Hematology, First Affiliated Hospital of Shantou University Medical College, Shantou, Guangdong, China; 3 Joint Shantou International Eye Center of Shantou University and the Chinese University of Hong Kong, Shantou, Guangdong, China; 4 Department of Thyroid Surgery, Shantou Central Hospital, Shantou, Guangdong, China

**Keywords:** non-alcoholic fatty liver disease, alanine aminotransferase, high-density lipoprotein cholesterol, ratio, cross-sectional study, Chinese adults

## Abstract

**Background:**

Non-alcoholic fatty liver disease (NAFLD) is increasingly recognized as a major public health concern, with rising prevalence worldwide. The alanine aminotransferase to high-density lipoprotein cholesterol (ALT/HDL-C) ratio has emerged as a potential marker of metabolic dysfunction, but its association with NAFLD remains underexplored, particularly in Chinese populations. This study aimed to investigate the independent and nonlinear association between the ALT/HDL-C ratio and NAFLD in Chinese adults.

**Methods:**

This was a secondary analysis of a cross-sectional dataset. A total of 1,592 Chinese adults aged 40–70 years who underwent health checkups were included. NAFLD was diagnosed via abdominal ultrasound. The ALT/HDL-C ratio was calculated and analyzed as both a continuous and categorical variable (tertiles). Multivariable logistic regression, restricted cubic spline (RCS) analysis, and receiver operating characteristic curves were used to assess the association and predictive performance of the ALT/HDL-C ratio for NAFLD.

**Results:**

The prevalence of NAFLD was 61.1%. After full adjustment, each one standard deviation increase in the ALT/HDL-C ratio was associated with a 79% higher odds of NAFLD (odds ratio [OR] = 1.79, 95% CI: 1.39–2.31, *p* < 0.001). A significant positive trend was observed across ALT/HDL-C tertiles (p for trend < 0.001). RCS analysis revealed a non-linear association (*p* for non-linearity = 0.002). Subgroup analyses showed a stronger association in females (OR = 3.89, *p* < 0.001) than in males (OR = 1.66, *p* < 0.001). However, no significant association was observed among adults aged ≥ 60 years (OR = 1.49, 95% CI 0.90–2.48, *p* = 0.125). The ALT/HDL-C ratio demonstrated superior predictive performance (area under the curve = 0.710) compared to ALT or HDL-C alone.

**Conclusion:**

The ALT/HDL-C ratio is independently and nonlinearly associated with NAFLD in Chinese adults, with a particularly strong association in non-elderly individuals and women. This simple and readily available biomarker shows promise for enhancing NAFLD risk stratification in middle-aged adults, while its utility in the elderly population may be limited and requires further investigation.

## 1 Introduction

Non-alcoholic fatty liver disease (NAFLD) has become a pervasive public health concern, affecting approximately 30% of the global adult population and standing as a leading cause of chronic liver disease (1). This worsening epidemiological profile is primarily attributable to the rising burden of metabolic risk factors—namely overweight, obesity and type 2 diabetes mellitus (2, 3). Beyond its intrinsic burden as a spectrum of liver conditions ranging from simple steatosis to cirrhosis, NAFLD significantly elevates the risk of debilitating extrahepatic complications, particularly cardiovascular disease, which stands as the primary cause of mortality in this patient population (1, 2, 4). This multifaceted risk profile underscores the critical importance of early detection. Implementing preventive strategies, such as weight management and physical activity, at the initial stages of steatosis can effectively halt or reverse disease progression (5).

A key barrier to early intervention lies in the limitations of current diagnostic approaches (6, 7). The reference standard, liver biopsy, is impractical for population-level screening due to its invasive nature and potential complications (8). Although non-invasive imaging modalities like vibration-controlled transient elastography offer promising alternatives, their accessibility in routine primary care remains limited (8). Commonly employed blood-based markers, including isolated liver enzyme measurements, often demonstrate inadequate performance for reliably identifying early-stage NAFLD, creating a pressing need for more robust and accessible tools (9). Consequently, developing simple, cost-effective biomarkers from readily available laboratory parameters is paramount to improving risk stratification and enabling timely management in broader clinical settings. This pursuit has led to the development of numerous non-invasive serum models and scores (10–12).

Alanine aminotransferase (ALT) is a well-established marker of hepatocellular injury and is commonly elevated in NAFLD (9, 13, 14). However, its specificity is limited, as ALT levels can be influenced by various factors including muscle mass, sex, and medications. High-density lipoprotein cholesterol (HDL-C), on the other hand, reflects lipid metabolism and has anti-inflammatory and antioxidant properties (15, 16). Low HDL-C levels are frequently observed in individuals with metabolic dysfunction, including NAFLD. The ALT/HDL-C ratio integrates hepatic injury and lipid metabolism into a single metric. The ALT/HDL-C ratio integrates hepatic injury and lipid metabolism into a single metric. Recent studies have suggested that this ratio may serve as a promising biomarker for NAFLD (17–19), with superior predictive performance compared to either ALT or HDL-C alone (17). However, current evidence is largely constrained by the use of selective cohorts, an absence of comprehensive subgroup analyses, and limited exploration of potential non-linear dose–response relationships. Moreover, data on the ALT/HDL-C ratio in Chinese populations remain scarce.

In this study, we aimed to investigate the association between the ALT/HDL-C ratio and NAFLD in a large Chinese cohort. We further explored whether this association is modified by demographic or metabolic factors, and evaluated the ratio’s predictive utility for NAFLD risk.

## 2 Methods

### 2.1 Study design and data source

This was a secondary analysis of a publicly available dataset originally collected and published by Yan et al. (20). The original dataset was publicly available in the Dryad repository (21). The parent study was a single-center, cross-sectional study conducted at Wuhan Union Hospital, China, between January 2020 and November 2021. It aimed to investigate the association between the fat-to-muscle ratio (FMR) and NAFLD. Ethical approval was granted by the Institutional Review Board of Tongji Medical College, Huazhong University of Science and Technology. The requirement for informed consent was waived due to the retrospective nature of the study. As the dataset is de-identified and publicly available via the Dryad repository, no additional ethical approval was required for this secondary analysis.

### 2.2 Study population

The original study enrolled adults aged 40–79 years who underwent routine health checkups. We applied the same exclusion criteria as the parent study: excessive alcohol consumption (>210 g/week for men, >140 g/week for women), known liver disease (viral, autoimmune, or drug-induced), acute illness, renal insufficiency (estimated glomerular filtration rate <60 mL/min/1.73 m^2^), active cancer, corticosteroid use, or missing biochemical or questionnaire data. After exclusions, 1,592 participants were included in the final analysis.

### 2.3 Data collection and variable definitions

Anthropometric measurements were performed by trained technicians. Body composition (weight, fat mass, muscle mass) was assessed using a multi-frequency bioelectrical impedance analyzer (Tsinghua Tongfan BCA-2A, China). The FMR was calculated as total fat mass divided by total muscle mass. Body mass index (BMI) was calculated as weight (kg) divided by height squared (m^2^). Seated blood pressure was measured twice after at least 10 min of rest using an electronic sphygmomanometer (Panasonic EW3106, China), and the average was recorded.

Fasting venous blood samples were analyzed in the central laboratory. Biochemical parameters included ALT, aspartate aminotransferase (AST), total cholesterol (TC), triglycerides (TG), HDL-C, low-density lipoprotein cholesterol (LDL-C), uric acid (UA), fasting blood glucose (FBG), and platelet count (PLT). The exposure variable of interest, the ALT/HDL-C ratio, was calculated as serum ALT (U/L) divided by HDL-C (mmol/L).

NAFLD was diagnosed based on abdominal ultrasonography (Philips IU22) performed by trained technicians. Participants with hepatic steatosis in the absence of other liver diseases were classified as having NAFLD. Diabetes was defined as self-reported diagnosis or use of glucose-lowering medication; hypertension was similarly defined.

### 2.4 Statistical analysis

Participants were categorized into tertiles based on the ALT/HDL-C ratio: tertile 1 (*n* = 528, < 15.09), tertile 2 (*n* = 533, 15.09–250.89) and tertile 3 (*n* = 531, ≥ 250.90). Categorical variables were expressed as frequencies (percentages) and compared using chi-square tests. Normally distributed continuous variables were presented as mean ± standard deviation and compared using one-way ANOVA; non-normally distributed variables were reported as median (interquartile range, IQR) and compared using the Kruskal–Wallis test.

Multivariable logistic regression was used to assess the association between the ALT/HDL-C ratio and NAFLD, expressed as odds ratios (ORs) with 95% confidence intervals (CIs). Three models were constructed: Model 1: Unadjusted; Model 2: Adjusted for sex, age, tobacco use, alcohol use, hypertension, and diabetes; Model 3: Further adjusted for BMI, FMR, PLT, AST, UA, FBG, TC, TG, LDL-C, SBP, and DBP. Restricted cubic splines (RCS) with four knots were used to examine potential non-linearity in the relationship between the ALT/HDL-C ratio and NAFLD. The likelihood ratio test was used to assess non-linearity. Subgroup analyses were performed to evaluate effect modification by sex, age (≤60 vs. >60 years), BMI (<25 vs. ≥25 kg/m^2^), tobacco use, alcohol use, hypertension, and diabetes. Interaction terms were tested using logistic regression. Receiver operating characteristic (ROC) curves were plotted to evaluate the predictive performance of the ALT/HDL-C ratio for NAFLD. The area under the curve (AUC) was compared with that of ALT and HDL-C alone using DeLong’s test.

All analyses were performed using SPSS version 27.0 and R software version 4.0.5. A two-sided *p* < 0.05 was considered statistically significant.

## 3 Results

### 3.1 Characteristics of the population by ALT/HDL-C ratio tertiles

A total of 1,592 patients were included in the study, with males constituting 72.1% of the cohort. The median ALT/HDL-C ratio was 19.77 (IQR: 12.93–31.33). The diagnosis of non-alcoholic fatty liver disease (NAFLD) was established in 973 cases, yielding a prevalence rate of 61.1%. The baseline characteristics of the study population stratified by tertiles of the ALT/HDL-C ratio are presented in Table 1. Across ascending tertiles, there was a progressive rise in the proportion of male patients and those with hypertension or diabetes, alongside increased rates of tobacco and alcohol consumption. Concurrently, higher tertile groups showed significantly elevated values for BMI, FMR, AST, ALT, FBG, UA, TC, TG, LDL-C, SBP, and DBP, along with a decline in both the proportion of elderly patients and HDL-C levels. NAFLD prevalence was also markedly greater in the upper tertiles. In contrast, no significant differences were detected in PLT levels across the groups.

**Table 1:** Baseline characteristics of the study population by tertiles of ALT/HDL-C ratio.

Variables	Tertile 1 (*n* = 530)	Tertile 2 (*n* = 532)	Tertile 3 (*n* = 530)	*P*
Male (*n*, %)				<0.001
Female	273 (51.70)	110 (20.64)	61 (11.49)	
Male	255 (48.30)	423 (79.36)	470 (88.51)	
Age ≥ 60 years (%)	204 (38.64)	187 (35.08)	138 (25.99)	<0.001
Tobacco use (%)				<0.001
No	418 (79.17)	326 (61.16)	310 (58.38)	
Yes	110 (20.83)	207 (38.84)	221 (41.62)	
Alcohol use (%)				<0.001
No	409 (77.46)	342 (64.17)	321 (60.45)	
Yes	119 (22.54)	191 (35.83)	210 (39.55)	
Hypertension (%)				<0.001
No	286 (54.17)	184 (34.52)	179 (33.71)	
Yes	242 (45.83)	349 (65.48)	352 (66.29)	
Diabetes (%)				<0.001
No	424 (80.30)	348 (65.29)	322 (60.64)	
Yes	104 (19.70)	185 (34.71)	209 (39.36)	
BMI (kg/m^2^)	23.80 (22.30,25.60)	23.80 (22.30,25.60)	23.80 (22.30,25.60)	<0.001
FMR	0.39 (0.32,0.50)	0.39 (0.32,0.50)	0.39 (0.32,0.50)	<0.001
PLT (10^9^/L)	207.50 (176.00,241.25)	207.50 (176.00,241.25)	207.50 (176.00,241.25)	0.638
ALT (U/L)	13.00 (11.00,16.00)	13.00 (11.00,16.00)	13.00 (11.00,16.00)	<0.001
AST (U/L)	18.00 (15.00,20.00)	18.00 (15.00,20.00)	18.00 (15.00,20.00)	<0.001
UA (umol/L)	315.70 (261.17,381.32)	315.70 (261.17,381.32)	315.70 (261.17,381.32)	<0.001
FBG (mmol/L)	4.91 (4.56,5.37)	4.91 (4.56,5.37)	4.91 (4.56,5.37)	<0.001
TC (mmol/L)	4.58 ± 0.99	4.37 ± 1.04	4.47 ± 1.23	0.007
TG (mmol/L)	1.04 (0.78,1.47)	1.04 (0.78,1.47)	1.04 (0.78,1.47)	<0.001
HDL-C (mmol/L)	1.35 ± 0.33	1.08 ± 0.23	0.92 ± 0.21	<0.001
LDL-C (mmol/L)	2.75 ± 0.82	2.61 ± 0.88	2.63 ± 0.95	0.023
SBP (mmHg)	128.00 (120.00,139.00)	128.00 (120.00,139.00)	128.00 (120.00,139.00)	0.005
DBP (mmHg)	80.00 (72.00,88.00)	80.00 (72.00,88.00)	80.00 (72.00,88.00)	<0.001
ALT/HDL-C ratio	10.85 (8.45,12.90)	10.85 (8.45,12.90)	10.85 (8.45,12.90)	<0.001
NAFLD (%)				<0.001
No	314 (59.47)	194 (36.40)	111 (20.90)	
Yes	214 (40.53)	339 (63.60)	420 (79.10)	

Data are shown as mean ± standard deviation (SD) or median (IQR) for continuous variables and proportions (%) for categorical variables.

ALT/HDL-C ratio, alanine aminotransferase to high-density lipoprotein cholesterol ratio; BMI, body mass index; FMR, fat-to-muscle ratio; PLT, platelet count; ALT, alanine aminotransferase; AST, aspartate aminotransferase; UA, serum uric acid; FBG, fasting blood glucose; TC, total cholesterol; TG, triglycerides; HDL-C, high-density lipoprotein cholesterol; LDL-C, low-density lipoprotein cholesterol; SBP, systolic blood pressure; DBP, diastolic blood pressure; NAFLD, non-alcoholic fatty liver disease.

### 3.2 Association between ALT/HDL-C ratio and NAFLD

Table 2 summarizes the association between the ALT/HDL-C ratio and NAFLD. In the unadjusted Model 1, a higher ALT/HDL-C ratio was significantly associated with increased odds of NAFLD (OR = 2.833, 95% CI: 2.321–3.456, *p* < 0.001). After adjusting for sex, age, smoking, alcohol use, hypertension, and diabetes in Model 2, the association remained significant (OR = 2.338, 95% CI: 1.909–2.864, *p* < 0.001). In the fully adjusted Model 3, which additionally included BMI, FMR, PLT, AST, UA, FBG, TC, TG, LDL-C, SBP, and DBP, the association persisted (OR = 1.790, 95% CI: 1.388–2.308, *p* < 0.001).

**Table 2:** Association between ALT/HDL-C ratio and NAFLD.

ALT/HDL-C ratio	Model 1		Model 2		Model 3	
	OR (95%CI)	*P*	OR (95%CI)	*P*	OR (95%CI)	*P*
ALT/HDL-C ratio (per 1 SD)	2.833 (2.321–3.456)	<0.001	2.338 (1.909–2.864)	<0.001	1.790 (1.388–2.308)	<0.001
ALT/HDL-C ratio (Tertile)						
Tertile 1	Ref		Ref		Ref	
Tertile 2	2.564 (2.002–3.284)	<0.001	2.072 (1.588–2.702)	<0.001	1.614 (1.199–2.713)	0.002
Tertile 3	5.552 (4.230–7.287)	<0.001	4.332 (3.222–5.284)	<0.001	2.389 (1.637–3.486)	<0.001
*P* for trend		<0.001		<0.001		<0.001

Model 1: unadjusted.

Model 2: sex, age, tobacco use, alcohol use, hypertension and diabetes.

Model 3: adjusted for variables in Model 2 plus body mass index, fat-to-muscle ratio, platelet count, aspartate aminotransferase, uric acid, fasting blood glucose, total cholesterol, triglycerides, low-density lipoprotein cholesterol, systolic blood pressure and diastolic blood pressure.

ALT/HDL-C ratio, alanine aminotransferase to high-density lipoprotein cholesterol ratio; NAFLD, non-alcoholic fatty liver disease; OR, odds ratio; CI, confidence interval.

When analyzed categorically by tertiles, a significant positive trend was observed across increasing ALT/HDL-C ratio categories (*p* for trend < 0.001). In unadjusted analysis, compared with the lowest tertile (T1), the odds ratios for NAFLD were 2.564 (95% CI: 2.002–3.284, *p* < 0.001) for T2 and 5.552 (95% CI: 4.230–7.287, p < 0.001) for T3. This graded association persisted after full adjustment (p for trend < 0.001), with adjusted ORs of 1.614 (95% CI: 1.199–2.713, *p* = 0.002) for T2 and 2.389 (95% CI: 1.637–3.486, *p* < 0.001) for T3.

**Figure 1:**
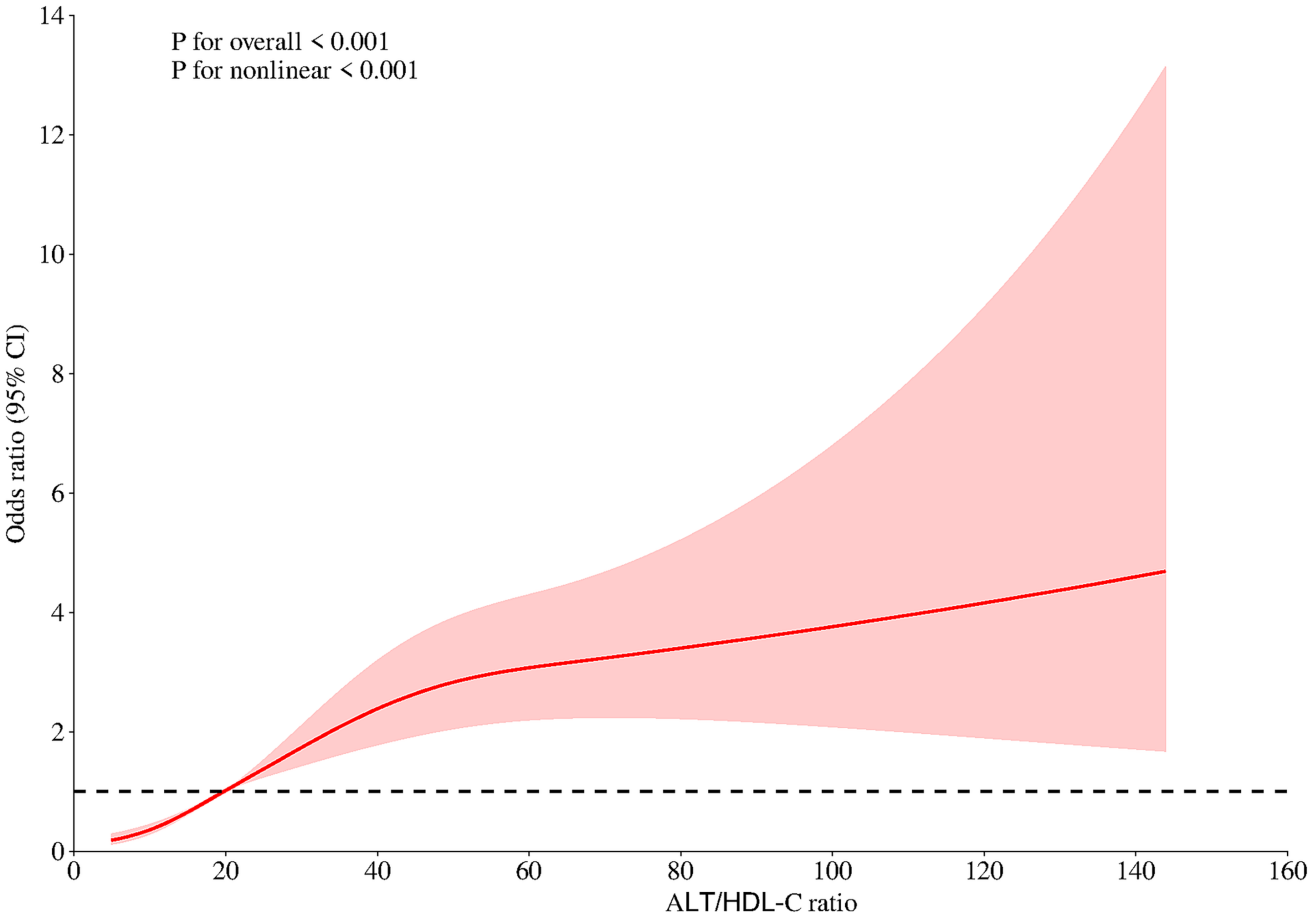
Restricted cubic spline models. Restricted cubic spline models with multivariable-adjusted associations were adopted to demonstrate dose–response associations between ALT/HDL-C ratio and NAFLD. ALT/HDL-C ratio, alanine aminotransferase to high-density lipoprotein cholesterol ratio; NAFLD, non-alcoholic fatty liver disease; OR, odds ratio; CI, confidence interval.

The present study investigated the potential nonlinear relationship between the ALT/HDL-C ratio and NAFLD using smoothed curve fitting. The results revealed a significant nonlinear association between the ALT/HDL-C ratio and the prevalence of NAFLD (*p* for non-linear = 0.002; Figure 1).

### 3.3 Subgroup analyses

**Figure 2:**
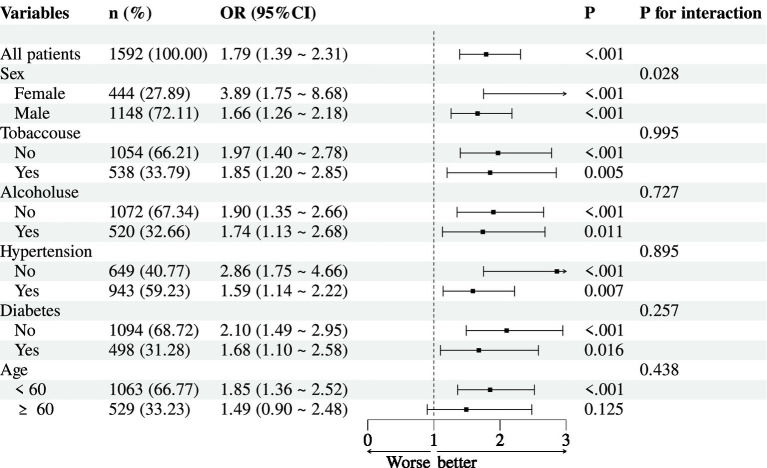
Subgroup analyses for association between ALT/HDL-C ratio (per 1 SD) and NAFLD.

We performed subgroup analyses to evaluate potential effect modification in the association between the ALT/HDL-C ratio (per 1 SD increase) and NAFLD risk (Figure 2). A significant interaction was observed for sex (*P*-interaction = 0.028). The association was stronger in females (OR = 3.89, 95% CI: 1.75–8.68, *p* < 0.001) than in males (OR = 1.66, 95% CI: 1.26–2.18, *p* < 0.001). No significant interactions were found for tobacco use (*P*-interaction = 0.995), alcohol use (*P*-interaction = 0.727), hypertension (*P*-interaction = 0.895), diabetes (*P*-interaction = 0.257), or age (<60 vs. ≥60 years, P-interaction = 0.438). However, within each subgroup, the ALT/HDL-C ratio remained significantly associated with NAFLD risk, except among participants aged ≥60 years, where the association was non-significant (OR = 1.49, 95% CI: 0.90–2.48, *p* = 0.125).

### 3.4 Predictive performance of ALT/HDL-C ratio for NAFLD

**Figure 3:**
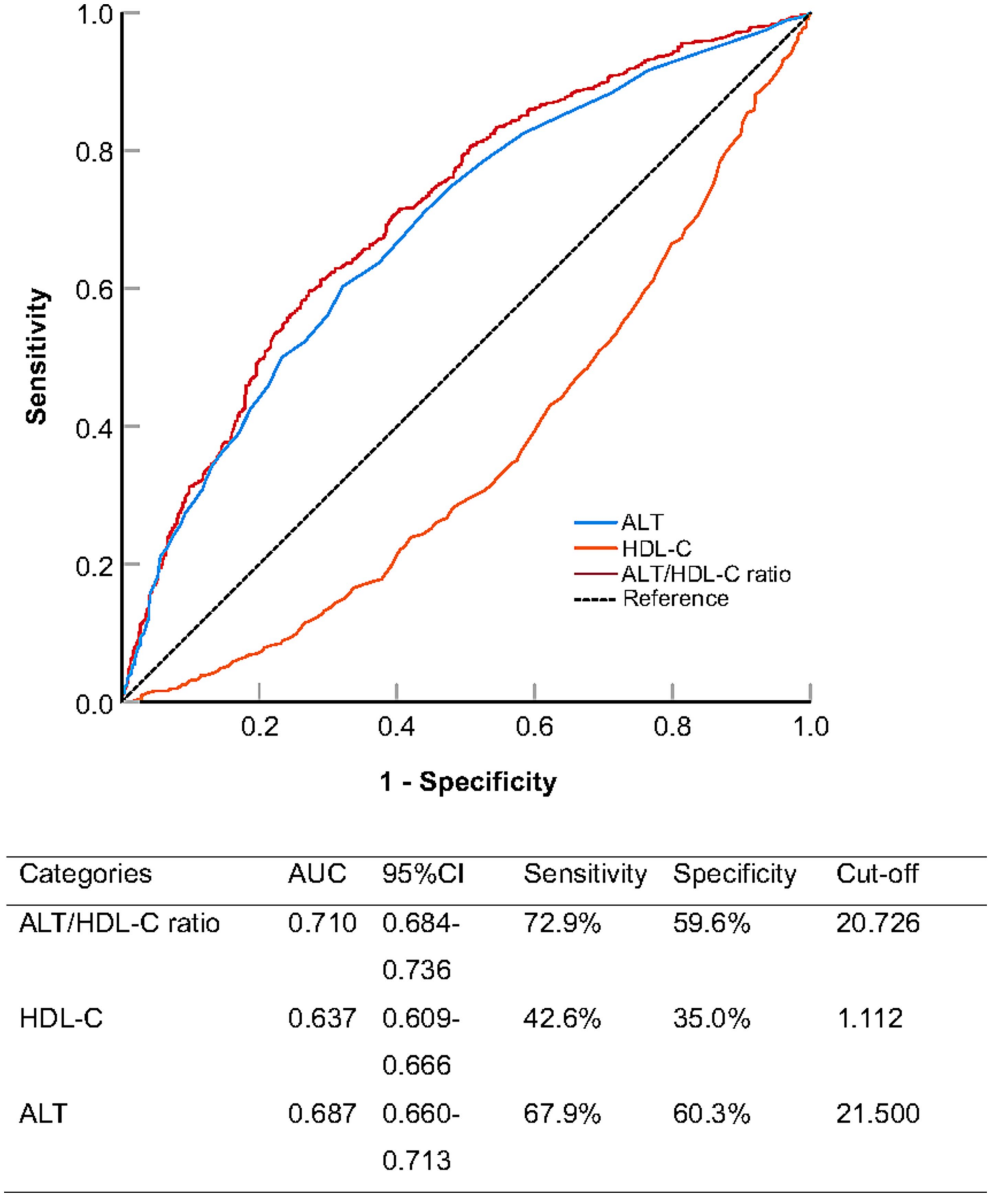
Receiver operating characteristic analysis comparing the predictive ability of alanine aminotransferase to high-density lipoprotein cholesterol ratio (ALT/HDL-C ratio), alanine aminotransferase (ALT), and high-density lipoprotein cholesterol (HDL-C) for non-alcoholic fatty liver disease.

To evaluate the predictive performance of the ALT/HDL-C ratio for NAFLD, we conducted ROC curve analysis and compared it with ALT and HDL-C alone (Figure 3 and Table 3). The area under the curve (AUC) for the ALT/HDL-C ratio was 0.710 (95% CI: 0.684–0.736), which was significantly higher than that of ALT alone (AUC = 0.687, *p* < 0.001) and HDL-C alone (AUC = 0.637, *p* < 0.001).

**Table 3:** Paired comparison of ROC curves (DeLong’s test).

Categories	Difference of AUC	Standard error	95%CI	*z*-value	*P*
ALT/HDL-C ratio vs. ALT	0.023	0.162	0.011–0.036	3.579	<0.001
ALT/HDL-C ratio vs. HDL-C	0.347	0.167	0.299–0.395	14.194	<0.001

ROC, receiver operating characteristic; AUC, area under curve; CI, confidence interval; ALT/HDL-C ratio, alanine aminotransferase to high-density lipoprotein cholesterol ratio; ALT, alanine aminotransferase; HDL-C, high-density lipoprotein cholesterol.

At the optimal cut-off value of 20.726, the ALT/HDL-C ratio achieved a sensitivity of 72.9% and a specificity of 59.6%, demonstrating a better balance between sensitivity and specificity compared to ALT and HDL-C individually.

## 4 Discussion

In this cross-sectional study involving 1,592 participants from a single-center cohort, we systematically investigated the relationship between the ALT/HDL-C ratio and the prevalence of NAFLD. Our results clearly demonstrated that elevated ALT/HDL-C ratio was significantly associated with increased risk of NAFLD, exhibiting a strong dose–response relationship across tertiles. This association remained robust after comprehensive adjustment for demographic characteristics, metabolic parameters, and lifestyle factors. Notably, the ratio demonstrated superior predictive performance for NAFLD compared to either ALT or HDL-C alone, with an AUC of 0.710 (95% CI: 0.684–0.736). Subgroup analyses revealed important effect modifications, showing a stronger association in female participants and a non-significant association in elderly individuals (≥60 years). Furthermore, we identified a significant nonlinear relationship between ALT/HDL-C ratio and NAFLD risk, adding nuance to our understanding of this association. Taken together, these data supported the ALT/HDL-C ratio as a simple, inexpensive and clinically accessible biomarker for identifying individuals at increased risk of NAFLD.

Our findings contribute to a growing body of evidence supporting the utility of combined biomarkers in assessing NAFLD risk (10–12). The ALT/HDL-C ratio integrates information from both hepatic injury and lipid metabolism pathways, providing a more comprehensive assessment of metabolic health than either parameter alone. Only three studies to date have specifically investigated the association between the ALT/HDL-C ratio and NAFLD (17–19). Qiu et al. (17) were the first to demonstrate the clinical utility of this ratio, showing that it had superior predictive performance for NAFLD compared to using ALT or HDL-C alone in a Korean population. Building on this, Cao et al. (18) reported a similar positive correlation in lean Chinese individuals and highlighted its potential as a non-invasive biomarker for early NAFLD detection. Further expanding these findings, Xuan et al. (19) demonstrated that the ALT/HDL-C ratio was independently associated not only with the presence of NAFLD but also with the severity of hepatic fibrosis. Our study reinforces these previous findings while adding several novel aspects, including the examination of nonlinear relationships, comprehensive subgroup analyses, and detailed assessment of potential effect modifications. The consistency of our results with previous literature across different populations strengthens the validity of ALT/HDL-C ratio as a reliable marker for NAFLD risk assessment.

Several interconnected mechanisms may account for the association between the ALT/HDL-C ratio and hepatic fat accumulation. First, ALT serves as a well-established biomarker of hepatocellular injury and inflammation (13, 14); its elevation often precedes the onset of steatosis and reflects enhanced hepatic *de novo* lipogenesis (9, 22), which is frequently driven by excessive carbohydrate intake and insulin resistance (23, 24). Second, a low level of HDL-C is not merely a passive correlate but an active contributor to lipotoxicity: impaired reverse cholesterol transport reduces the export of free cholesterol from the liver, thereby promoting endoplasmic reticulum stress and overproduction of very-low-density lipoprotein (16, 25). Given the central role of oxidative stress in the initiation and progression of NAFLD (26), it is reasonable to propose that the antioxidant properties of HDL-C may contribute to the pathophysiology of this condition (15). Third, both elevated ALT and reduced HDL-C are closely linked to adipose tissue insulin resistance (27, 28), which exacerbates lipolysis and increases the delivery of free fatty acids to the liver (29). Consequently, the ALT/HDL-C ratio integrates two complementary pathological pathways—hepatocellular damage and dysregulated lipid metabolism—into a single quantitative measure, offering a comprehensive indicator of hepatic metabolic stress.

A pivotal finding of our study was the significant interaction by sex (*P*-interaction = 0.028). The association between the ALT/HDL-C ratio and NAFLD was markedly stronger in females (OR = 3.89, 95% CI: 1.75–8.68) than in males (OR = 1.66, 95% CI: 1.26–2.18). This disparity suggests that the pathophysiological pathways linking this ratio to NAFLD may differ between sexes. Several biological and hormonal factors could explain this observation. Premenopausal women are relatively protected from NAFLD, largely due to the beneficial effects of estrogen on lipid metabolism and insulin sensitivity (30). Consequently, when NAFLD develops in women, it might be driven by a more profound metabolic disturbance (31), which the ALT/HDL-C ratio could be particularly sensitive in capturing. ALT may serve as a simple diagnostic tool to identify insulin-resistant subjects only in women (32). The ratio, integrating hepatic injury (ALT) and a key marker of metabolic health (HDL-C), may thus be a more powerful indicator of a disrupted metabolic state in females. In contrast, the higher baseline prevalence of NAFLD and related metabolic risk factors in males might attenuate the relative strength of the association. This finding underscores the necessity of considering sex-specific strategies when utilizing biomarkers for NAFLD risk assessment.

Another critical observation was the non-significant association observed in participants aged 60 years and older (OR = 1.49, 95% CI: 0.90–2.48, *p* = 0.125), despite a strong association in those under 60. This age-related discrepancy may be attributed to several factors. The aging process is associated with sarcopenia, changes in body composition, and alterations in lipid profiles (33–35), which might alter the relationship between conventional biomarkers and NAFLD. Furthermore, older populations often have a higher prevalence of comorbidities and polypharmacy, which could confound the association. Consequently, the ALT/HDL-C ratio, which integrates hepatic injury and lipid metabolism, appears to be a robust indicator of the “typical” metabolic dysfunction driving NAFLD in middle-aged adults, but may be less sensitive to the more complex and heterogeneous pathophysiology in the elderly. Therefore, our data suggest that the ALT/HDL-C ratio is a powerful tool for NAFLD risk stratification primarily in the non-elderly adult population. For elderly patients, clinicians should be aware of its limited sensitivity and may need to prioritize other assessment methods.

Our study adds several novel aspects to the existing literature. First, we identified a significant nonlinear relationship between the ALT/HDL-C ratio and NAFLD risk. Second, we conducted extensive subgroup analyses that revealed important sex-specific differences in the association, providing new insights into potential effect modifications. Third, we established an optimal cut-off value of 20.726 with clinically relevant sensitivity (72.9%) and specificity (59.6%), offering a practical threshold for clinical implementation. The ALT/HDL-C ratio represents a simple, cost-effective, and readily available biomarker that could be easily incorporated into routine clinical practice for NAFLD screening. Its superiority over individual parameters makes it particularly valuable for risk stratification in primary care settings where specialized diagnostic tools may be unavailable. The identified cut-off value provides a concrete threshold for clinicians to identify high-risk individuals who might benefit from further diagnostic evaluation or intensive lifestyle interventions.

Despite these important findings, several limitations should be acknowledged. First and foremost, the diagnosis of NAFLD was based on abdominal ultrasonography rather than the histological gold standard of liver biopsy. While ultrasound is a practical and widely accepted tool for non-invasive detection of hepatic steatosis in large cohorts, it has inherent limitations, including operator dependency and reduced sensitivity for mild steatosis (typically requiring >20–30% hepatic fat infiltration). Furthermore, ultrasonography cannot differentiate simple steatosis from the more progressive form of non-alcoholic steatohepatitis (NASH) or assess the degree of liver fibrosis. This diagnostic approach may have led to misclassification, particularly of participants with early-stage NAFLD. It is important to note that such non-differential misclassification would most likely bias our results toward the null hypothesis, suggesting that the true association between the ALT/HDL-C ratio and NAFLD might be stronger than what we observed. Second, the cross-sectional design precludes any causal inference regarding the relationship between the ALT/HDL-C ratio and NAFLD. Third, as a single-center study, our findings may have limited generalizability to other populations with different ethnic compositions or healthcare settings. Finally, although we adjusted for a comprehensive set of potential confounders, residual confounding from unmeasured factors such as detailed dietary patterns, physical activity levels, or genetic predisposition cannot be entirely excluded.

In conclusion, the ALT/HDL-C ratio is independently, dose-dependently and non-linearly associated with NAFLD in Chinese adults, particularly those under 60 years of age. Its superior discriminative ability in this large at-risk demographic, coupled with minimal additional cost and universal availability, renders it an attractive tool for first-line NAFLD screening in resource-limited primary-care settings. Future research should focus on developing and validating age-specific biomarker panels for accurate NAFLD detection in the elderly.

## Data Availability

The datasets presented in this study can be found in online repositories. The names of the repository/repositories and accession number(s) can be found: the data that support the findings of this study are openly available in Dryad at: https://datadryad.org/dataset/doi:10.5061/dryad.7d7wm3809.
